# Third Dentition

**Published:** 1891-10

**Authors:** 


ARTICLE VI.

### THIRD DENTITION.

Captain William Bruce, aged eighty-two, living at 1025 Valencia street, San Francisco, has just cut his third set of teeth. The Captain is an old and well-known resident of the city, and his case has occasioned much interest.

For more than twenty years, Captain Bruce says, his gums were compelled to do the work of mastication. Between two and three years ago he began to be troubled by sharp pains in his gums, which continued at intervals day after day. He was at a loss to account for this, nor were the medical men whom he consulted able to give any explanations or relief. Finally, one bright morning, on making a careful examination, he was astonished to find the point of a white molar introducing itself for service from his tender gums.

Gradually it forced its way out, and soon began to assume respectable proportions. This extraordinary and unexpected visitor explained, in a measure, the suffering which its owner had been compelled to endure; and but for the fact that the pains continued after the tooth had fully appeared, he would have concluded that it was the only one with which he was to be blessed.

### MORE OF THEM COME.

The growing process, however, was much slower than in the case of teeth-cutting with children, but it proved to be just as sure. Another molar on the opposite side of the mouth next popped out; then came a couple of incisors, then the canine teeth and bicuspids, till at last he has two shining rows of teeth as perfect, if not quite as large, as any owned by men but half his years.

The teeth made their appearance at intervals of from one to two months duration, and, as may naturally be supposed, much merriment was occasioned among the friends of the old gentleman when word was passed around among them that "the Captain's got another tooth."

An examination shows the teeth to be firmly set in the gums, if not in their proper foundation in the jaws, and that they will, in all human probability, serve him well for the remainder of his years.

TRULY A PHENOMENON.

"The cutting of a third set of teeth, or third dentition, as it is professionally termed," said a well-known dentist recently, "has occurred only at very rare intervals. Occasionally medical or dental journals have noted cases, but the only authentic ones on record are quoted in some of the old English works on dentistry. Of the known cases, all occurred at an advanced age, and but two are recorded in which the persons got complete sets of teeth. One was that of a woman ninety-seven years of age, and the other that of a man of eighty-six years. One or two cases have been heard of in Eastern cities, but investigations have failed to prove their correctness, the actual trouble being nothing but the remains of old stumps working their way through the gums."

Captain Bruce, while being well pleased with the arrival of his new and serviceable companions, is at the same time somewhat annoyed at the prominence he has gained by their coming.—*Items of Interest.*

---

### Communications.

---

### A CORRECTION.

---

*Editor American Journal of Dental Scieuce:*

